# Computational Fluid Dynamics Support for Fontan Planning in Minutes, Not Hours: The Next Step in Clinical Pre-Interventional Simulations

**DOI:** 10.1007/s12265-021-10198-6

**Published:** 2021-12-27

**Authors:** Petter Frieberg, Nicolas Aristokleous, Pia Sjöberg, Johannes Töger, Petru Liuba, Marcus Carlsson

**Affiliations:** 1 Department of Clinical Sciences Lund, Clinical Physiology, Skåne University Hospital, Lund University, Lund, Sweden; 2 Department of Mechanical & Manufacturing Engineering, University of Cyprus, Nicosia, Cyprus; 3 Department of Clinical Sciences Lund, Pediatric Heart Center, Skåne University Hospital, Lund University, Lund, Sweden; 4 Laboratory of Clinical Physiology, NHLBI, National Institutes of Health, Bethesda, MD USA

**Keywords:** Patient-specific CFD, Fontan procedure, Hemodynamics, Congenital heart disease, Children, Fontan circulation

## Abstract

Computational fluid dynamics (CFD) modeling may aid in planning of invasive interventions in Fontan patients. Clinical application of current CFD techniques is however limited by complexity and long computation times. Therefore, we validated a “lean” CFD method to magnetic resonance imaging (MRI) and an “established” CFD method, ultimately aiming to reduce complexity to enable predictive CFD during ongoing interventions. Fifteen Fontan patients underwent MRI for CFD modeling. The differences between lean and established approach, in hepatic and total flow percentage to the left pulmonary artery (%LPA), power loss and relative wall shear stress area were 1.5 ± 4.0%, -0.17 ± 1.1%, -0.055 ± 0.092 mW and 1.1 ± 1.4%. Compared with MRI, the lean and established method showed a bias in %LPA of -1.9 ± 3.4% and -1.8 ± 3.1%. Computation time was for the lean and established approach 3.0 ± 2.0 min and 7.0 ± 3.4 h, respectively. We conclude that the proposed lean method provides fast and reliable results for future CFD support during interventions.

**Graphical abstract:**

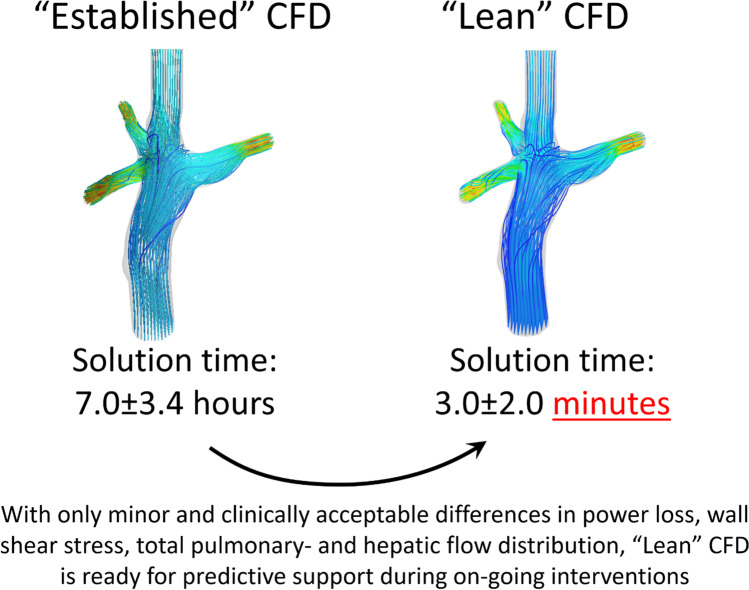

## Introduction

Congenital heart disease (CHD) is the most common class of major congenital malformations and is the leading cause of mortality from birth defects [1]. The reported prevalence of CHD in the general population varies between 8 and 10 per 1000 live births [2]. Modern surgical and medical care have significantly extended life expectancy and this leads to increasing number of adult CHD patients, thereby increasing the needs for health resources in this patient group [1, 3].

Single ventricle (SV) includes a wide range of complex univentricular CHD where children typically require 2 to 3 surgical palliations, aiming to achieve lung perfusion via connection between the systemic caval veins and the pulmonary artery branches, called total cavopulmonary connection (TCPC) or “Fontan” circulation.

The first palliation is often performed neonatally and consists of either surgical or transcatheter shunt between a major systemic central vessel or the right ventricle and the pulmonary artery. At the second stage (“bidirectional Glenn”), the superior vena cava (SVC) is surgically anastomosed to the right pulmonary artery (RPA). At the third stage (“TCPC”), blood in the inferior vena cava (IVC) is directed into the pulmonary arteries via an extracardiac GoreTex™ conduit [4, 5]. While early outcomes are generally acceptable, severe adverse events may occur in the long-term including protein-losing enteropathy, declining functional status, increased pulmonary vascular resistance, exercise intolerance, heart failure, and hepatic and renal dysfunction [6, 7]. These complications may lead to redo surgery or transcatheter interventions. These procedures are often complex and would therefore greatly benefit from predictive computer simulations of treatment outcome. Ideally simulations should be used earlier to predict the results of Glenn to TCPC surgery and guide the initial surgery to optimize the Fontan circulation.

Currently, simulations using computational fluid dynamics (CFD) offer the possibility to model the Glenn and TCPC circulation and can provide measures of flow and how these would be affected by interventions that change the geometry. Furthermore, advanced hemodynamic measures such as power loss (${\dot{E}}_{loss}$) [8] and wall shear stress (WSS) [9] can be obtained from CFD and may further the understanding of pathophysiology in the Fontan circulation.

However, clinical use of CFD is still limited. A major reason is the complex methods, which involves the use of advanced software, powerful computers, and interaction between clinicians, medical physicists, and engineers [10]. Other reasons found are sources of uncertainty that accumulate during the modeling process from image artifacts, noise, and inherent limitations of the image acquisition, to simulation assumptions and simplifications [11]. Trusty et al. provided a detailed description of this time-consuming procedure, with 60 h required for simulation of one surgical option at one physiologic condition [10].

Our goal is to have a clinically integrated and lean CFD framework by which clinicians and engineers can interactively perform interventional planning and get CFD results on pre-prepared models, all within minutes. Ultimately, we aim to realize a framework that could enable near real-time predictive CFD support adjacent to the operating room during ongoing interventions.

We have previously proposed a simplified “lean” method to reduce the complexity in this process so that more centers can adopt CFD in clinical routine [12], but it has not been validated and compared with established CFD approaches. One further simplification to reduce computation time is to assume steady flow and ignore the pulsatile nature of venous flows [13], but how this simplification affects lean CFD results has not been previously examined.

Therefore, we aimed to validate both a clinically feasible lean CFD approach and an established CFD approach using identical boundary conditions, against flow measurements from magnetic resonance imaging (MRI). Next, we aimed to compare the lean and established approach for additional hemodynamic measurements such as hepatic flow distribution, wall shear stress, and power loss. Furthermore, we aimed to verify previous findings [13] on whether steady (time-averaged) flow is an acceptable simplification compared to pulsatile flow computed using both the lean and the established CFD solver. Finally, we aimed to record the required effort in user- and computation time to produce reliable CFD results using the proposed methods.

## Methods

### Study Population and MR Imaging

Patients with SV (*n* = 15, median age 6.7, range 2.3 to 17 years, 6 females) with either bidirectional Glenn (*n* = 6) or TCPC (*n* = 9) were included in the study. The Glenn patients were prospectively included in a research protocol and imaged at a 1.5 T Siemens Aera scanner (Siemens Healthineers, Erlangen, Germany). The TCPC patients were retrospectively included in a previous study and imaged at a 1.5 T Philips Achieva scanner (Philips Healthcare, Best, The Netherlands) [12]. Cine images were acquired using a steady-state free precession (SSFP) sequence (typical parameters TR/TE/flip angle: 2.9 ms/1.5 ms/60°, slice thickness 5 mm, in-plane resolution 1.2 mm × 1.2 mm). Two-dimensional phase contrast MRI (2D PC-MRI) flow measurements were acquired using a velocity encoded fast field echo sequence (typical parameters TR/TE/flip angle: 10 ms/6.5 ms/15° in-plane resolution 1.2 mm × 1.2 mm). Flow measurements of the SVC, the IVC/TCPC conduit, the pulmonary artery branches, and pulmonary veins were typically acquired using velocity encoding 80 cm∙s^−1^ and for the aorta 200 cm∙s^−1^. Image analysis was done using the freely available software Segment (Medviso AB, Lund, Sweden, http://segment.heiberg.se) [14]. The study was approved by the Regional Ethical Review Board in Lund, Sweden. Adult patients gave written informed consent. For patients under 18 years of age, written informed consent was given by their parents. Patient characteristics are shown in Table 1.

**Table 1:** Patient characteristics and summarized hemodynamic data from MRI. *BSA* body surface area, *LPA* left pulmonary artery, *%PFD*_*LPA*_ fraction of total pulmonary blood to LPA, *VCS* vena cava superior, *VCI* vena cava inferior, *RPA* right pulmonary artery, *Flow* BSA indexed time averaged flow, *wPI* weighted pulsatile index, *APC* aortopulmonary collateral flow (total pulmonary vein flow — total pulmonary artery flow)

Patient group	Age (years)	BSA (m^2^)	%PFD_LPA_	wPI	VCS flow (l/min/m^2^)	VCI flow (l/min/m^2^)	RPA flow (l/min/m^2^)	LPA flow (l/min/m^2^)	APC flow (l/min/m^2^)
TCPC (*n* = 9) mean ± *SD* (range)	9.2 ± 5.6 (3–17)	1.1 ± 0.43 (0.56–1.7)	38 ± 11% (17–53%)	46 ± 14% (29–72%)	0.82 ± 0.19 (0.60–1.2)	1.2 ± 0.51 (0.36–2.3)	1.2 ± 0.39 (0.57–1.8)	0.71 ± 0.41 (0.12–1.6)	1.1 ± 0.69 (0.14–2.4)
Glenn (*n* = 6) mean ± *SD* (range)	2.8 ± 0.26 (2.3–3.1)	0.56 ± 0.03 (0.52–0.60)	43 ± 6.7% (35–53%)	48 ± 8.6% (36–61%)	1.5 ± 0.26 (1.2–1.9)	1.3 ± 0.22 (0.96–1.7)	0.85 ± 0.17 (0.65–1.1)	0.64 ± 0.16 (0.42–0.82)	1.1 ± 0.35 (0.58–1.4)
All (*n* = 15) mean ± *SD* (range)	6.6 ± 5.4 (2.3–17)	0.90 ± 0.43 (0.52–1.7)	40 ± 10% (17–53%)	47 ± 12% (29–72%)	1.1 ± 0.39 (0.69–1.9)	1.3 ± 0.42 (0.36–2.3)	1.1 ± 0.36 (0.57–1.8)	0.68 ± 0.33 (0.12–1.6)	1.5 ± 0.26 (0.14–2.4)

### Surface Model Reconstruction

**Fig. 1:**
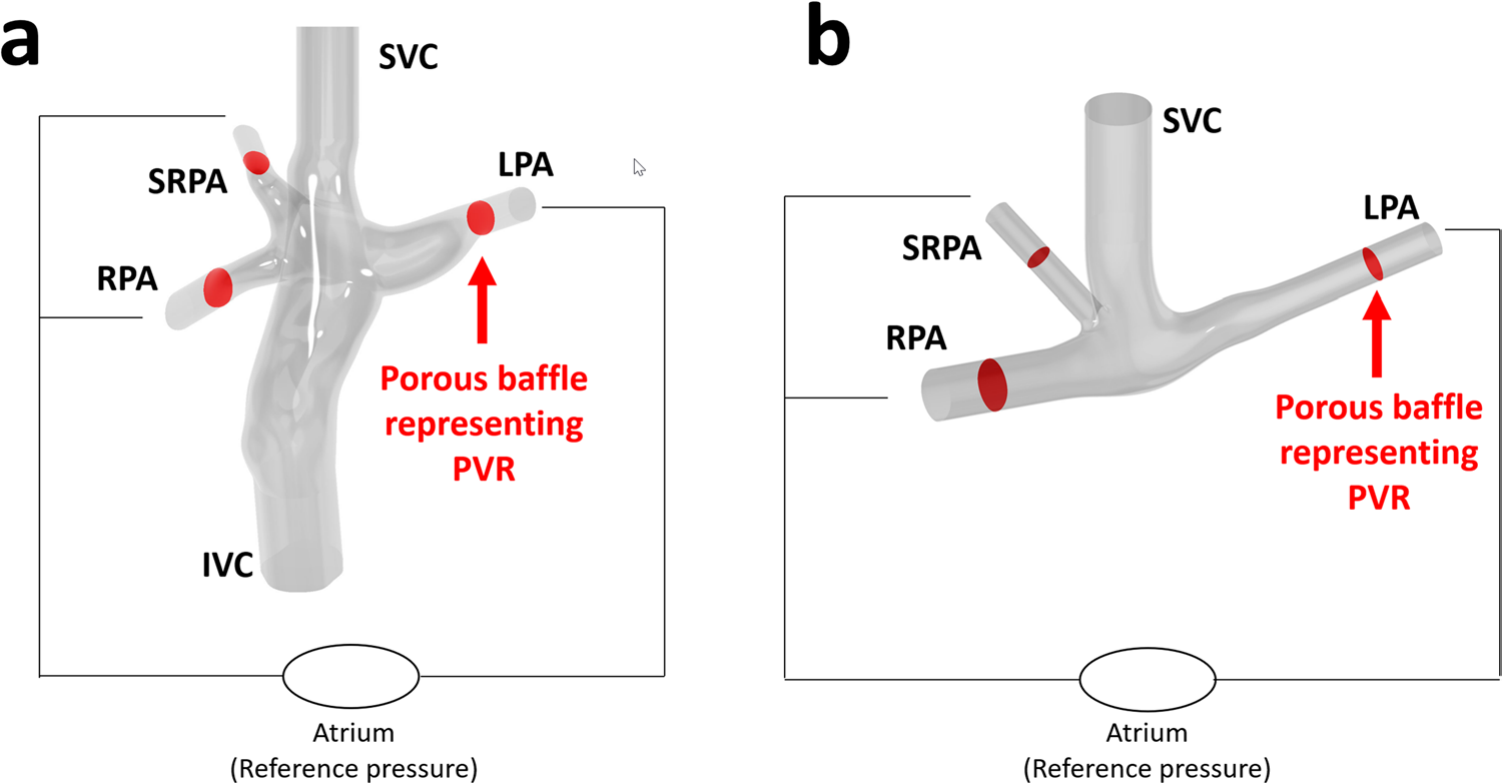
Two representative surface models of Fontan (**a**) and Glenn (**b**) structures, with limbs namely the inferior vena cava (IVC), superior vena cava (SVC), left pulmonary artery (LPA), right pulmonary artery (RPA), and superior right pulmonary artery (SRPA). Red arrows highlight porous baffle locations representing linear PVR, proximal to the distal common pressure potential of the atrium

Patient-specific 3D models of the proximal Glenn/TCPC anastomosis were constructed in Creo Parametric (PTC, Boston, MA, USA). The geometry was created by importing MRI segmentation curves created in Segment into Creo Parametric, where geometry was created on the curve boundaries. Examples of TCPC and Glenn models are shown in Fig. 1.

### Numerical Approach

In this study, two different commercial CFD software packages and patient specific results from MRI were used to obtain hemodynamic and time resource parameters. The following comparisons were made under identical boundary conditions in CFD:

- Pulmonary flow distribution to the left pulmonary artery obtained from the lean and established CFD were compared with results from MRI as the reference standard.
- Pulmonary- and hepatic flow distribution to the left pulmonary artery, power loss, and normalized wall shear stress obtained from the lean and established CFD were compared under steady and pulsatile inlet boundary conditions.
- Time required for meshing, simulation set-up, and solving were compared for the lean and established CFD.

### Computational Fluid Dynamics

The numerical solution was obtained using two commercial CFD packages. For the lean CFD, we used a solver Simcenter FloEFD for Creo (Siemens EDA, Wilsonville, OR, USA). FloEFD is embedded in the user interface of Creo Parametric and uses the immersed boundary method which is well known and has been previously used in Fontan CFD simulations [15, 16] As established CFD we used as solver STAR-CCM + (v2019.1, Siemens PLM Software, Plano, TX, USA), whose numerical core is based on the finite volume method (FVM).

Each new patient set-up was based on a typical “Fontan template” with pre-populated fields with non-patient-specific values for inlet flows, outlet boundaries, measurement of hemodynamic parameters, and rheology in the software. These are out-of-the-box capabilities that we found was the most time-efficient approach in both methods.

All simulations and setup thereof were performed by the same user who is an expert operator well acquainted with both softwares.

### Boundary Conditions

The simulations used the following common modeling assumptions:

- blood was considered as an incompressible non-Newtonian fluid using the Carreau model with viscosity *η*(*γ*) according to Eq. (1) with density = 1060 kg∙m^−3^, zero shear viscosity *η*_0_ = 0.033 kg∙m^−1^ˑs^−1^, infinite shear viscosity *η*_∞_ = 0.006109, time constant *λ* = 3.34 s, and power law index *n* = 0.3035 [17].
  1 $$\eta \left(\gamma \right)={\eta }_{\infty }+\left({\eta }_{0}-{\eta }_{\infty }\right){\left[1+{\left(\gamma \lambda \right)}^{2}\right]}^{\frac{n-1}{2}}$$
- rigid vessel walls
- laminar flow
- patient specific mass flow rates from MRI were applied at each inlet
- zero pressure at the outlets representing the common pressure of the atrium

The models included a proxy of pulmonary vascular resistance (PVR) based on MRI flows. This allows the pulmonary artery flow to change following future anatomic interventions, assuming PVR will not change in the short term. The used model of resistance satisfies the fundamental equation:

2 $$\Delta p=R \cdot F$$

where Δ*p* is the transpulmonary gradient, *R* is the pulmonary vascular resistance, and *F* is the pulmonary artery flow. Linear porosity satisfies this equation and is ubiquitous among commercial CFD packages. Porosity was used to model constant values of *R*, assigned to porous baffles near the outlets (Fig. 1). Single ventricle patients often present with aortopulmonary collaterals (APC), which can be quantified with MRI as the difference between pulmonary vein flow and pulmonary artery flow [18]Compared to the proximal pulmonary artery flow, APC flow increases the total pulmonary flow *F* with a factor *k*:

3 $$k=\frac{Pulmonary vein flow}{Pulmonary artery flow}$$

which can be obtained from MRI for each patient and for each lung. Instead of physically including APC flow in the simulations, the constant pulmonary vascular resistance *R* was multiplied with patient specific values of *k*, as a proxy for the effect of the increased pulmonary flow *F* due to APC [12].

In the established CFD, the solution was considered converged when continuity and velocity-scaled residuals dropped below < 10^−5^. In pulsatile simulations, 4000 iterations per cycle were used. In the lean CFD, the default internal algorithms were used. User-specified measurements of power loss, total and hepatic mass flow to the left pulmonary artery were monitored for convergence during the solution process.

In all pulsatile simulations inlet velocity waveforms acquired by MRI were decomposed in 15 harmonics using Matlab R2019a (The MathWorks Inc., Natick, MA, USA).

In TCPC patients, results were reported from the 6th cycle to reach a cyclic steady-state in the hepatic flow from IVC to the pulmonary arteries. In Glenn patients, results were reported from the 3d cycle to reach a cyclic steady-state.

### Meshing

Systematic mesh convergence studies were performed for both methods. More details on meshing have been described previously [19, 20]. In the established CFD we found mesh-independent results with tetrahedral element sizes of approximately 1/15 the average pulmonary artery diameter, corresponding to 2–3% of the IVC diameter. On average, this represented 1.6 million elements in the TCPC patients and 1.1 million elements in the Glenn patients. Elements were constructed semi-automatically in ICEM CFD v19.2 (Ansys Inc., Canonsburg, PA, USA). The established CFD program reported no invalid elements, typical aspect ratios of 0.8 to 1.0 for 85% of the elements (min aspect ratio typically > 0.45) and no skewness angles > 75° (max skewness angle typically < 65°).

In the immersed boundary method used by the lean method, the software automatically generates hexagonal elements within the minimal volume required to completely immerse the model in elements, of which the vast majority will be perfectly hexagonal. The immersed boundary method then “cuts” any corners that protrude outside the computational domain using well-understood correction methods [16]. Mesh-independent results were found with approximately 150,000 elements for TCPC patients and approximately 140,000 for the Glenn patients. This corresponded to a mesh size of approximately 10% of the IVC diameter.

### Statistics

Statistical analysis was performed using GraphPad (v8.0, La Jolla, CA, USA). Linear regression was used to analyze correlation between calculated results and the reference method. Accuracy and precision were calculated according to Bland–Altman analysis as mean ± *SD* of the measured difference against the mean of the results. Results with *p* < 0.05 were considered significant.

### Hardware

A workstation with an 8-core Intel i7700k processor @ 5.0 GHz was used for all steady-state simulations (lean and established CFD), and for the pulsatile simulations using the lean method.

The pulsatile CFD simulations in the established CFD were performed on a computation server with 44 compute cores (2 × 22-core Intel Xeon Gold 6152, Intel Corporation, Santa Clara, CA, USA), of which 32 cores were used for the CFD simulations.

### Hemodynamic Parameters Compared Between the Methods

1. Pulmonary flow distribution to the left pulmonary artery (%PFD_LPA_):
  $${\mathrm{\%}PFD}_{LPA}=\frac{{Q}_{LPA}}{{Q}_{LPA}+{Q}_{RPA}}\bullet 100\mathrm{\%}$$
  where ${Q}_{LPA}$ and ${Q}_{RPA}$ are the left and right pulmonary artery flow, respectively.
2. Hepatic flow distribution to the left pulmonary artery (%HFD_LPA_):
  $${\mathrm{\%}HFD}_{LPA}=\frac{{Q}_{IVC to LPA}}{{Q}_{IVC}}\bullet 100\mathrm{\%}$$
  where ${Q}_{IVC to LPA}$ and ${Q}_{IVC}$ are the flow from inferior vena cava directed to the left pulmonary artery and the inferior vena cava flow, respectively.
3. Power loss $$({\dot{E}}_{\mathrm{loss}})$$:
  $${\dot{E}}_{loss}=\sum_{inlets A}\int (p+\frac{1}{2}\rho {v}^{2})v\bullet dA-\sum_{outlets A}\int (p+\frac{1}{2}\rho {v}^{2})v\bullet dA$$
  where $p$ is the static pressure, $\rho$ is the blood density, $v$ is the velocity, and $A$ is the cross-section area of all inlets and outlets.
4. Wall shear stress, WSS (${\tau }_{\mathrm{w}}$):
  $${\tau }_{\mathrm{w}}=\mu \frac{\partial \mathrm{u}}{\partial \mathrm{y}}$$
  where *μ* is the dynamic viscosity and $\frac{\partial \mathrm{u}}{\partial \mathrm{y}}=\dot{\gamma }$ is the rate of shear.
5. Time average wall shear stress (TAWSS):
  $$TAWSS=\frac{1}{T}{\int }_{0}^{T}\left|{\tau }_{\mathrm{w}}\right|dt$$
  where *T* is the physical time of one heartbeat and *τ*_w_ is the local wall shear stress.
6. Pulsatile index (PI) [21]:
  $$PI=\frac{{Q}_{max}-{Q}_{min}}{2\times {Q}_{avg}}\times 100\mathrm{\%}$$
  where ${Q}_{max},{Q}_{min}$, and ${Q}_{avg}$ are the maximum, minimum, and average flow rates.
7. Weighted pulsatile index (wPI) to represent an overall pulsatility level of the Fontan connection [21]:
  $$wPI=\sum_{i=1\dots n}{PI}_{i}\times {C}_{i}$$
  where ${C}_{i}$ is the relative flow split of vessel *i* and ${C}_{i}=\frac{{Q}_{i}}{{Q}_{mean}}$
8. Normalized wall shear stress area was calculated as the ratio of the area with WSS or TAWSS < 0.4 Pa, compared to the total vessel wall area. This measure has been suggested to be associated with increased risk of atherosclerosis [22] and has been previously reported in Fontan patients [23].

## Results

**Fig. 2:**
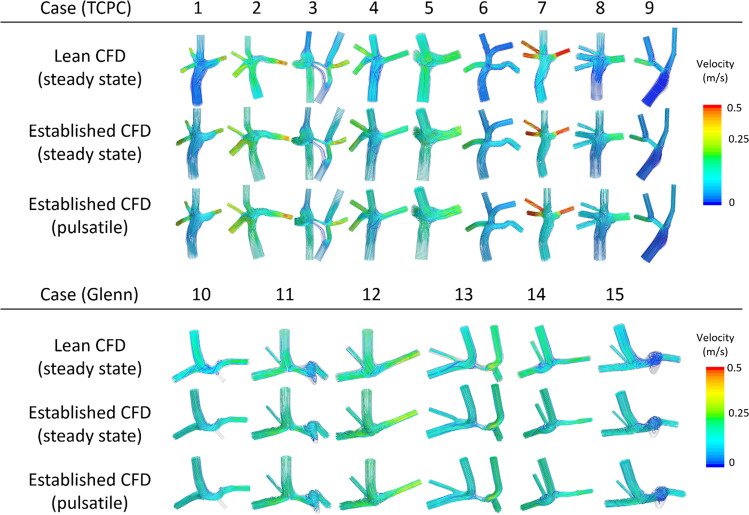
Flow streamlines for all investigated patients, TCPC (above), and Glenn (below). Results are shown from the lean CFD with steady-state flow, established CFD with steady-state flow, and established CFD with pulsatile flow

**Fig. 3:**
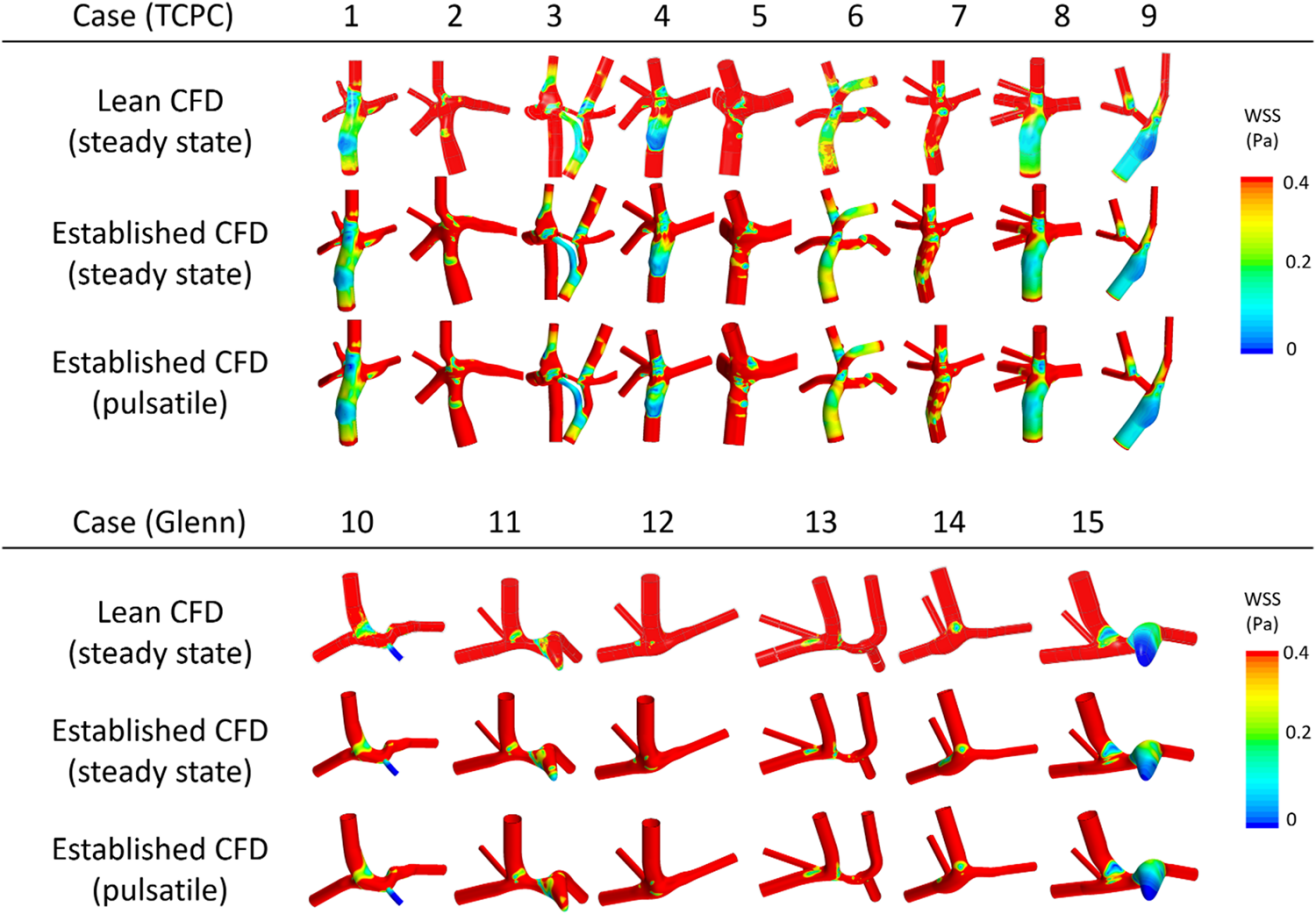
Contour plots of the wall shear stress (WSS) and time averaged WSS (pulsatile) for all investigated patients, TCPC (above), and Glenn (below). Results are shown from the lean CFD with steady-state flow, established CFD with steady-state flow, and established CFD with pulsatile flow

Qualitative comparison of velocity-coded flow patterns from the lean, steady established, and pulsatile established CFD showed essentially identical flow patterns in all patients (Fig. 2). Visual comparison of WSS from the lean, steady established, and pulsatile established CFD also showed very similar patterns (Fig. 3). Simulations showed a posterior vortex in the SVC/IVC junction in six of the nine TCPC patients.

**Fig. 4:**
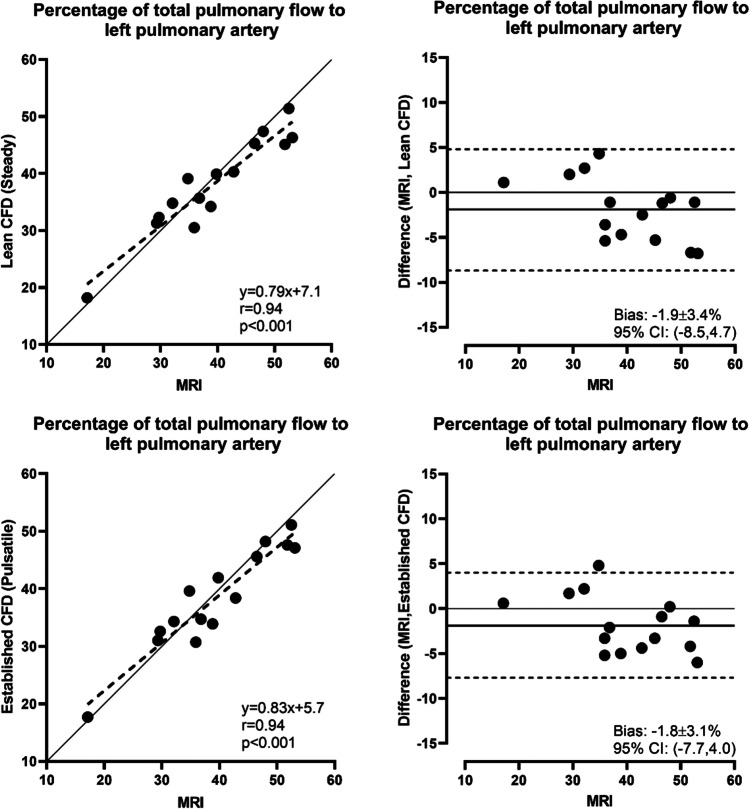
Top row: Linear regression (left) and Bland–Altman analysis (right) of pulmonary flow distribution to left pulmonary artery, comparing steady state lean CFD with MRI measurements (MRI). Bottom row: Linear regression results (left) and Bland–Altman plots (right) of pulmonary flow distribution to left pulmonary artery, comparing pulsatile established CFD with MRI measurements (MRI)

Linear regression analysis of %PFD_LPA_ from the steady lean and pulsatile established CFD both showed a correlation coefficient of 0.94 with the MRI data. Bland–Altman analysis of %PFD_LPA_ from the steady lean and pulsatile established CFD compared to MRI data showed a bias of -1.9 ± 3.4% for the lean CFD and -1.8 ± 3.1% for the established CFD (Fig. 4).

Among the Glenn patients, MRI results of %PFD_LPA_ were 43 ± 6.7%, compared with steady-state lean CFD and pulsatile established CFD of 40 ± 6.6% and 40 ± 6.2% respectively. CFD ${\dot{E}}_{\mathrm{loss}}$ were 0.51 ± 0.17 mW and 0.58 ± 0.21 mW respectively. CFD normalized WSS area were 11 ± 10% and 11 ± 9.9% respectively (Tables 3 and 4).

Among the TCPC patients, MRI results of %PFD_LPA_ were 38 ± 11%, compared with steady-state lean CFD and pulsatile established CFD of 37 ± 9.2% and 37 ± 9.5% respectively. CFD %HFD_LPA_ were 41 ± 22% and 43 ± 24% respectively. CFD ${\dot{E}}_{\mathrm{loss}}$ was 1.9 ± 1.6 mW and 1.9 ± 1.5 mW respectively. CFD normalized WSS areas were 29 ± 18% and 27 ± 17% respectively (Tables 3 and 4).

**Fig. 5:**
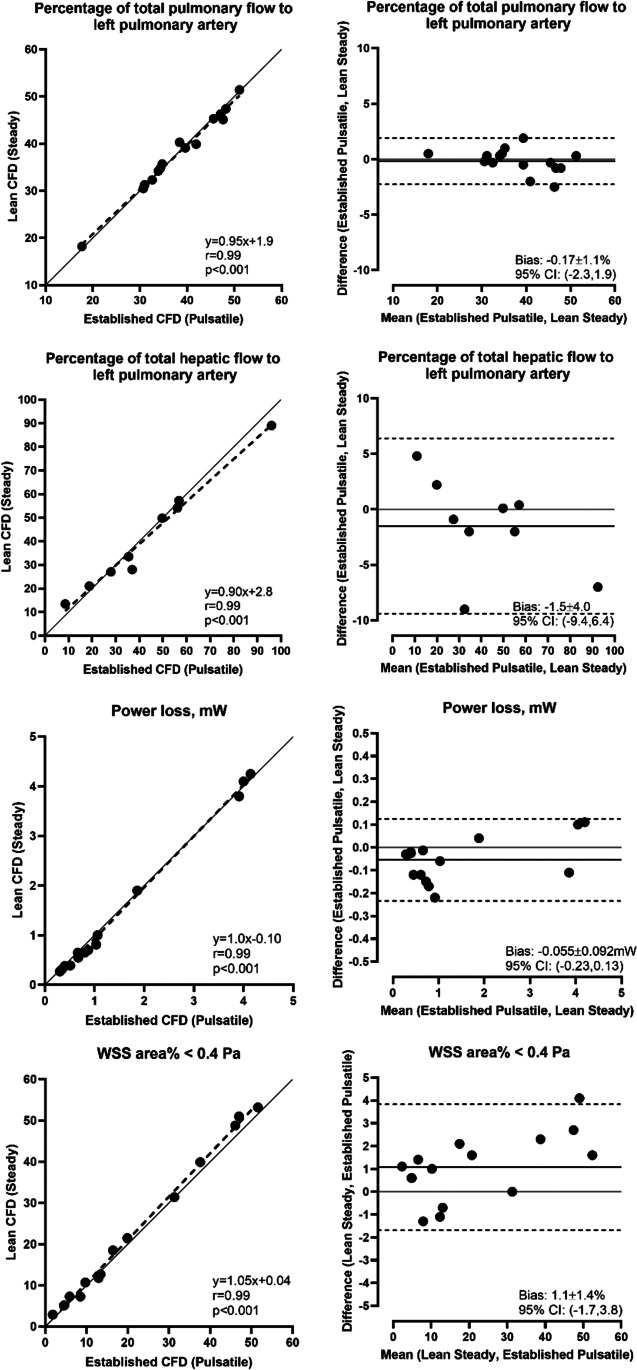
First row: Linear regression (left) and Bland–Altman analysis (right) of total flow distribution to left pulmonary artery, comparing steady state lean CFD with pulsatile established CFD. Second row: Linear regression (left) and Bland–Altman analysis (right) of hepatic flow distribution to left pulmonary artery, comparing steady state lean CFD with pulsatile established CFD. Third row: Linear regression (left) and Bland–Altman analysis (right) of power loss, comparing steady state lean CFD with pulsatile established CFD. Fourth row: Linear regression (left) and Bland–Altman analysis (right) of normalized WSS area, comparing lean steady state results with established pulsatile CFD results

Comparing the hypothetically least accurate solution (lean time-averaged) with the hypothetically most accurate solution (established pulsatile), Bland–Altman analysis of steady-state ${\dot{E}}_{\mathrm{loss}}$, %PFD_LPA_, %HFD_LPA_, and normalized WSS showed a bias of -0.055 ± 0.092 mW, -0.17 ± 1.1%, -1.5 ± 4.0%, and 1.1 ± 1.4%, respectively. Similarly, linear regression analysis of ${\dot{E}}_{\mathrm{loss}}$, %PFD_LPA_, and %HFD_LPA_ and normalized WSS showed correlation coefficients of 0.99 (Fig. 5).

**Fig. 6:**
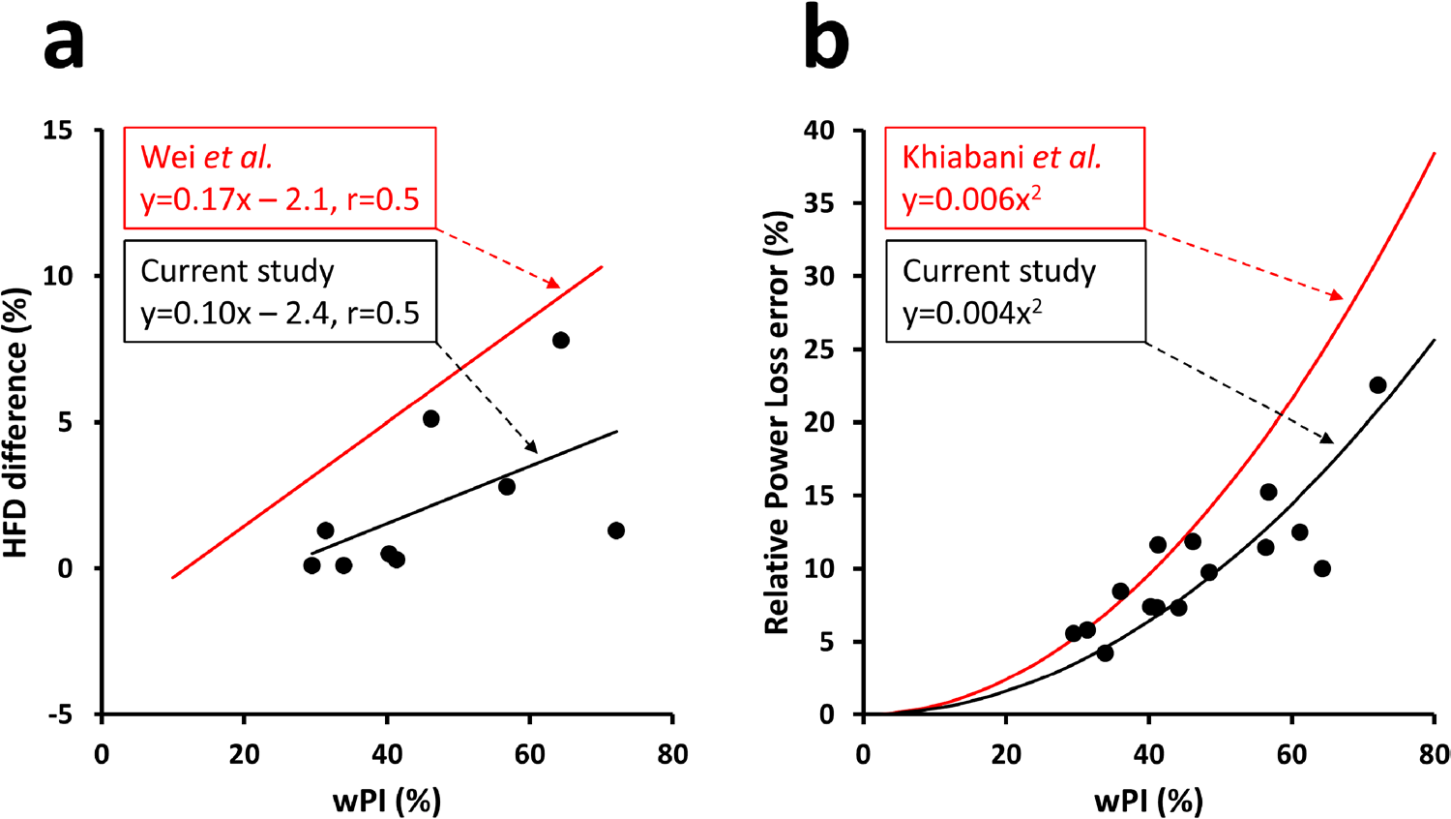
**a** Hepatic flow percentage (HFD) difference between lean steady-state and pulsatile simulations vs. weighted Pulsatile Index (wPI). **b** Relative power loss error between lean steady-state and pulsatile simulations vs. weighted pulsatile index (wPI)

The correlation of weighted pulsatility index (wPI) with the differences in %HFD_LPA_ and ${\dot{E}}_{\mathrm{loss}}$ between lean steady-state and pulsatile simulations are shown in Fig. 6.

In the lean CFD, meshing was part of the automatic solver process and took on average 10 ± 2.8 s per model. In the established CFD, semi-manual meshing took on average 21 ± 4.1 min per model. The process of setting up the CFD model, such as adding boundary conditions, preparation of measurements and convergence criteria took 15 ± 3 and 33 ± 5.6 min for the lean and established CFD, respectively (Table 2).

**Table 2:** User work time and computer solver time in minutes, mean ± *SD*

	User work time in minutes, mean ± *SD* (seconds in parenthesis)				Computer solver time in minutes, mean ± *SD* (hours in parenthesis)			
Patient group	Lean CFD		Established CFD		Lean CFD		Established CFD	
	Meshing	CFD settings	Meshing	CFD settings	Steady	Pulsatile	Steady	Pulsatile
TCPC	< 1	15 ± 3	23 ± 4.3	34 ± 3.4	3.7 ± 2.3	75 ± 29 (1.3 ± 0.5 h)	34 ± 12	570 ± 115 (9.5 ± 1.9 h)
Glenn	< 1	15 ± 3	19 ± 3.3	31 ± 7.7	2.0 ± 0.6	17 ± 3.3	11 ± 4.2	203 ± 65 (3.4 ± 1.1 h)
All	(10 ± 2.8 s)	15 ± 3	21 ± 4.1	33 ± 5.6	3.0 ± 2.0	52 ± 36	25 ± 15	421 ± 203 (7.0 ± 3.4 h)
					Workstation 8 cores			Server 32 cores

Among Glenn patients, solver times were for the steady-state lean and pulsatile established CFD 3.0 ± 2.0 min and 203 ± 65 min (3.4 ± 1.1 h), respectively. Among TCPC patients, solver times were for the steady-state lean and pulsatile established CFD 3.7 ± 2.3 min and 570 ± 115 min (9.5 ± 1.9 h), respectively (Table 2).

Additional results in terms of user- and solver time, as well as separately reported hemodynamic results for Glenn and TCPC patients using the lean and established steady-state and pulsatile CFD are shown in Tables 2, 3, and 4, respectively.

**Table 3:** Summarized steady state CFD results. Lean: CFD performed with the lean CFD platform. Established: CFD performed with the established CFD. *LPA* left pulmonary artery, *%PFD*_*LPA*_ fraction of total pulmonary blood to LPA, *%HFD*_*LPA*_ fraction of hepatic blood to LPA, *PL* power loss, *%WSS area* < *0.4 Pa* area fraction of wall shear stress < 0.4 Pa, *NA* not applicable

Patient group	Steady state CFD results							
	%PFD_LPA_		%HFD_LPA_		PL (mW)		%WSS area < 0.4 Pa	
	Lean CFD	Established CFD	Lean CFD	Established CFD	Lean CFD	Established CFD	Lean CFD	Established CFD
TCPC (*n* = 9) mean ± *SD* (range)	37 ± 9.2% (18–52%)	37 ± 9.5% (18–51%)	41 ± 22% (13–89%)	43 ± 23% (18–95%)	1.9 ± 1.6 (0.27–4.3)	1.8 ± 1.5 (0.27–4.0)	29 ± 18% (7.3–49%)	28 ± 17% (7.3–49%)
Glenn (*n* = 6) mean ± *SD* (range)	40 ± 6.6% (31–47%)	40 ± 6.3% (31–48%)	NA	NA	0.51 ± 0.17 (0.31–0.70)	0.53 ± 0.19 (0.30–0.78)	11 ± 10% (2.9–31%)	11 ± 9.4% (1.8–30%)
All (*n* = 15) mean ± *SD* (range)	38 ± 8.2% (18–52%)	38 ± 8.5% (31–51%)	41 ± 22% (13–89%)	43 ± 23% (18–95%)	1.3 ± 1.4 (0.27–4.3)	1.3 ± 1.3 (0.27–4.0)	22 ± 18% (2.9–49%)	21 ± 17% (1.8–49%)

**Table 4:** Summarized pulsatile CFD results. Mean values in TCPC patients from heartbeat 6 of 6, in Glenn patients from heartbeat 3 of 3. Lean: CFD performed with the lean CFD platform. Established: CFD performed with the established CFD. *LPA* left pulmonary artery, *%PFD*_*LPA*_ fraction of total pulmonary blood to LPA, *%HFD*_*LPA*_ fraction of hepatic blood to LPA, *PL* power loss, *%WSS area* < *0.4 Pa* area fraction of wall shear stress < 0.4 Pa, *NA* not applicable. *Mean WSS area cannot be computed in pulsatile CFD in the lean CFD platform

Patient group	Pulsatile CFD results						
	%PFD_LPA_		%HFD_LPA_		PL (mW)		%WSS area < 0.4 Pa (*)
	Lean CFD	Established CFD	Lean CFD	Established CFD	Lean CFD	Established CFD	Established CFD
TCPC (*n* = 9) mean ± *SD* (range)	37 ± 9.3% (18–51%)	37 ± 9.5% (18–51%)	42 ± 23% (5.6–89%)	43 ± 24% (8.6–96%)	2.1 ± 1.7 (0.30–4.5)	1.9 ± 1.5 (0.30–4.1)	27 ± 17% (5.9–47%)
Glenn (*n* = 6) mean ± *SD* (range)	40 ± 6.7% (31–48%)	40 ± 6.2% (31–48%)	NA	NA	0.57 ± 0.20 (0.35–0.72)	0.58 ± 0.21 (0.34–0.87)	11 ± 9.9% (4.5–31%)
All (*n* = 15) mean ± *SD* (range)	38 ± 8.3% (18–51%)	38 ± 8.5% (18–51%)	42 ± 23% (5.6–89%)	43 ± 24% (8.6–96%)	1.5 ± 1.5 (0.30–4.5)	1.4 ± 1.4 (0.34–4.1)	21 ± 17% (4.1–47%)

## Discussion

This study shows that the proposed lean CFD approach and the established CFD approach produced very similar results, but with vastly shorter solution times for the lean, steady-state simulations. Our findings indicate that steady-state simulations using the lean method can be performed in approximately 4% of the time required for achieving nearly identical results using the established method with pulsatile inflows, whereas previous findings of Khiabani et al. showed savings of approximately 50% [21].

While the results show that the lean CFD approach can reproduce in vivo measurements from MRI and compares well with established CFD, clinical research is needed before broad application in clinical routine to show that the CFD simulations are accurate in their prediction of outcome after surgery and catheter interventions. The first study comparing CFD with post-operative outcome was recently published by Trusty et al. and showed in a retrospective analysis of 12 patients (whereof 7 Fontan completion surgeries) a fairly large bias between predicted flows and outcome [24]. This may be explained by changes in the patient’s physiology, thereby changing the boundary conditions. Examples of physiological changes are patient’s growth as well as changes in oxygenation, collateral flow and cardiac output. Validation of the possibility to predict intervention outcome using the lean CFD approach is thus needed.

### Benefits of the Lean Method

The proposed “lean” numerical pipeline uses less computational resources and less user input and thus use less resources to create value compared to the established methods. The three major differences that we identified making the proposed lean CFD faster and “lean” compared to established CFD are:

1. In the immersed boundary method used by the lean method, the software automatically generates hexagonal elements and benefits from the numerical advantages that follow, since hexagonal elements are more efficient compared to tetrahedral elements. The established method uses tetrahedral elements which require greater user attention to the mesh density, mesh quality, and the distribution of element sizes near the center and walls of the model. Thus, the lean method can obtain a correct solution faster, with less user interaction and with fewer elements than the established method.
2. The lean method integrates CFD with the geometric editing tool, requiring 0 transfer of data between softwares. This significantly saves time when iterating designs.
3. The lean CFD method has a process-oriented workflow making the use of software easier.

While Glenn and Fontan procedures are not emergency procedures, performing faster CFD for interventional planning with less resources are inherently valuable even for elective surgery.

Regionalization and centralization are increasingly adopted by western countries to promote efficiency, reduce excess mortality, and health care expenses. Thus, a tertiary referral center will cover a large geographic area and reducing the number of outpatient visits is important. With lean CFD, there is enough time to perform predictive simulations even when the pre-interventional MRI is performed after the patient has arrived for elective surgery. This may be a time-efficient way to obtain updated information prior to the surgical intervention and can save time and cost of travel for the family.

In addition, some patients require catheter-based interventions between and after the major Fontan surgical stages. Lean CFD and its short solution times may aid in interventional decision-making based on acute findings during such procedures. If MRI and catheterization is performed under the same general anesthesia, time is short to update the calculations.

Our reported simulation times of less than 5 min mean that such simulations can be performed well within the timeframe of ongoing invasive interventions. These reported time savings are likely conservative since the lean simulations ran on an 8-core computer, whereas the pulsatile simulations of the established method ran using 32 cores on a dedicated server.

While it was shown that the lean CFD approach has computation times as short as 3.0 min in average, another benefit for interventional work is the integration of the lean solver and the computer aided design (CAD) software. There is no need to manually update the CFD model after a geometric update in the CAD software. While not explicitly demonstrated in this work, this is important for the total time required for predictions of interventions. Whereas segmenting a model from scratch takes approximately 1 h, modifying an already prepared model in CAD to represent an intervention can interactively be done in seconds or minutes. Thus, simulating for example a stent dilatation would only require a minor CAD change and an additional 3.0 min of meshing and computation (Table 2), given that all other boundary conditions remain unchanged. In comparison, using established CFD, the entire work would have to be done from scratch based on the updated geometry, with minimum lead time of 1 h and 19 min (steady-state simulation) according to the presented findings. This potentially makes the lean approach suitable for “live” predictive CFD support during ongoing interventions, whereas the established CFD approach is not very suitable since it takes too long time to generate new results.

In the lean CFD, the workflow in the user interface involves graphical interaction with the model and does not expose the user to complex details of CFD simulation, thus making it easier to use in a clinical setup. Many established CFD settings are available in the lean approach but are not mandatory for the user to interact with.

The lean CFD approach can thus be used in clinical studies aiming to demonstrate the effect of proposed anatomical interventions on blood flow and offers the possibility to do clinically integrated simulations of surgical or catheter-based interventions in patients with Glenn and Fontan circulations at the hospital without use of advanced computational facilities.

A user of predictive CFD in a clinical setting should still be an expert user with demonstrated skills in the tools and procedures involved and this does not differ in the investigated methods. We believe that it will be easier to have more expert users with the productivity benefits of the lean method and the rapid iterations it enables with the clinicians as this may increase the demand and utility of computational simulations.

### Hemodynamic Measurements

We used several hemodynamic parameters for comparison between the techniques, some used in clinical routine imaging, such as pulmonary flow distribution. Other measures, such as hepatic flow distribution, are clinically relevant but only available through more advanced techniques, e.g., 4D-flow MRI or CFD. We also compared more advanced hemodynamic measures (${\dot{E}}_{\mathrm{loss}}$, WSS) that have been used in previous studies of single ventricles and shown to be of interest but have not reached clinical practice [17, 23, 25]. One reason for the lack of clinical use is the complexity in obtaining these values. Thus, our lean approach to CFD in single ventricle patients may lead to increased availability and the possibility to use some of these advanced hemodynamic measures in larger cohorts and demonstrating the potential clinical value. We found a pulmonary distribution (%PFD_LPA_) of 40.1%, which is similar to 43% measured by Tang et al. in a previous study with a notably large cohort (*n* = 108) [26]*.* Our measurements for the hepatic distribution (%HFD_LPA_) were 43% on average, similar to 50% calculated by Wei et al. [23]. Moreover, we calculated a power loss (${\dot{E}}_{\mathrm{loss}}$) of 1.3 ± 1.4 mW and 1.4 ± 1.4 mW on average for steady and pulsatile simulations, respectively. These values are similar to previous studies, e.g., Baretta et al. found a power loss of 1.54 mW at rest and 4.8 mW during exercise [27]; Bove et al. calculated a range of 4 to 56 mW [28]; and Marsden et al. found 6.7 mW and 13.9 mW in two patients [29]. Normalized WSS area, which is the area of WSS below 0.4 Pa divided by the total surface area was similar for the lean CFD (22 ± 18%) and established CFD (21 ± 17%). These results were similar to a study of Wei et al. [23] that reported normalized WSS area of 22%.

### Pulsatile vs. Steady-State Simulations

We calculated time averaged venous flows as the mean flows from the flow curves obtained in MRI during the cardiac cycle at supine rest during free breathing. However, the flow patterns in our Glenn and Fontan patients are much less pulsatile compared to venous flow in healthy subjects (illustrated in the pulsatility index). As seen in Table 2, computation times are significantly shorter for time-averaged simulations compared to pulsatile flow simulation.

With respect to hepatic flow and pulsatility, Wei et al. [13] showed that “a large portion of the cases can use time-averaged BCs to save computational cost,” and we found that differences in our patients fell within the lower range of this study (Fig. 6a). Wei et al. showed that such differences are mainly determined by weighted pulsatility index (wPI) and the IVC angle. We speculate that the found differences between the studies could be due to low patient number and the relatively perpendicular IVC insertion angle relative to the pulmonary arteries in the current study (Fig. 2), whereas Wei et al. had a wider range of angles. If required, the lean approach can significantly shorten pulsatile TCPC simulations which were shown to take 1.3 ± 0.5 h whereas the established CFD required 9.5 ± 1.9 h on a much faster computer (Table 2). Additionally, our findings of the relationship between power loss and pulsatility were similar to the findings of Khiabani et al. [21] (Fig. 6b).

### Limitations

User dependence and limited spatial resolution with possible inaccuracies in the identification of the vessel anatomy and blood flow rate will affect the results. However, our validation used MRI which is the clinical reference standard for measurement of intrathoracic flow, and this showed low bias and variability. Further limitations are simplifications such as assumed rigid walls and inlet plug flow profiles, but these are established assumptions in research studies of patient-specific CFD simulations [8, 17, 30–32].

Aiming to predict changed pulmonary artery flows following interventions, previous studies have simulated more advanced patient specific physiology at the outlets in different ways. Multiple studies have used compliant Windkessel models of PVR and “lumped parameter” models of the complete cardiovascular system in the CFD loop [26, 33]. These methods require more patient data as input and are more resource intensive.

The demonstrated lean method does not predict pulse pressures since it relies on non-invasive MRI data. If catheterized pressures are available, such data could potentially be used with the lean method to include patient-specific linear PVR and atrial reference pressure, but this has not been validated with patient data or compared with results from advanced models such as proposed by Ahmed et al. [33].

Given the limitations of using fixed PVR, our simplified pulmonary circuit including the effects of precapillary aortopulmonary collateral (APC) flow correlated well with the pulmonary flow split measured in patients with a correlation coefficient of 0.94 for both the lean and established CFD (Fig. 4) and has been successfully used to predict outcome of interventions [12].

While this model can be considered a fully non-invasive and lean approach, it should be considered a proxy for resistance and cannot predict absolute patient-specific values of Fontan pressures. However, for the purposes of calculating power loss, wall shear stress, and total and hepatic pulmonary flow distribution, this is not a limitation since they are not based on absolute pressures.

## Conclusion

Using a lean CFD framework on a basic workstation can save significant amount of manual work and computing resources compared with an established CFD solver with comparable accuracy and precision in the results. Specifically, the computational work time of the steady-state lean CFD solver, including meshing, was on average 23% of computational work time of the steady-state established CFD and on average 4% of the computational work time of the pulsatile established CFD. This lean CFD approach may help more centers deploy CFD for hemodynamic assessment of Fontan patients in clinical practice.

In summary, the proposed lean method provides reliable and clinically usable results, in a time-effective manner that enables the possibility for predictive CFD support during interventions.

## Declarations

### Animal Studies

No animal studies were carried out by the authors for this article.
